# Whole-genome sequences of *Bipolaris bicolor*, *Curvularia hawaiiensis*, *Curvularia spicifera*, and *Exserohilum rostratum* isolated from rice in Burkina Faso, France, Mali, and Pakistan

**DOI:** 10.1128/MRA.00134-23

**Published:** 2023-10-09

**Authors:** Kouka Hilaire Kaboré, Abalo Itolou Kassankogno, Henri Adreit, Sebastien Ravel, Florian Charriat, Diariatou Diagne, Marc-Henri Lebrun, Didier Tharreau

**Affiliations:** 1 Université Paris-Saclay, INRAE, UR BIOGER, Palaiseau, France; 2 CIRAD, UMR PHIM, Montpellier, France; 3 CNRST/INERA, Laboratoire de Phytopathologie, Bobo-Dioulasso, Burkina Faso; 4 PHIM, Univ Montpellier, CIRAD, INRAE, IRD, Institut Agro Montpellier, Montpellier, France; 5 Laboratoire de Biologie Moléculaire Appliquée (LBMA)/Université des Sciences, des Techniques et des Technologies de Bamako (USTTB), Bamako, Mali; University of California, Riverside, Riverside, California, USA

**Keywords:** phytopathology, rice disease, fungi, population genetics, diagnostic tools, genotypic identification, molecular detection, rice brown spot, genomics, pathogenesis

## Abstract

Different fungal species of the *Pleosporaceae* family infect rice, causing similar symptoms. Reference genomic sequences are useful tools to study the evolution of these species and to develop accurate molecular diagnostic tools. Here, we report the complete genome sequences of *Bipolaris bicolor*, *Curvularia hawaiiensis*, *Curvularia spicifera,* and *Exserohilum rostratum*.

## ANNOUNCEMENT

Rice is a worldwide crop widely consumed in Asia and Africa. Many diseases affect rice production, with an estimated annual loss of 20% in yield (1). Different fungal species infect rice (2), some of which are not sufficiently well studied, in particular, fungi responsible for the rice brown spot diseases, *Bipolaris bicolor*, *Curvularia hawaiiensis*, *Curvularia spicifera,* and *Exserohilum rostratum* (3, 4). In the perspective of developing molecular diagnostic markers, the availability of their complete genomes is essential (5). In 2018, rice seeds and leaves with typical brown spot disease symptoms were collected in rice fields in Burkina Faso, France, Mali, and Pakistan. They were incubated for 5 to 7 days on wet filter paper at 25°C with a 12-h photoperiod. One conidium was isolated from each sample, giving rise to monospore isolates. Genomic DNAs extracted from these isolates were used to amplify glyceraldehyde-3-phosphate dehydrogenase and translation elongation factor 1 alpha (TEF1-a) genes using the primers GPD1/GPD2 (6) and TEF1-983/TEF1-2218 (7), respectively. These PCR products were sequenced, and the alignments of these sequences with sequences in databases showed a 100% identity to reference sequences from *B. bicolor*, *C. hawaiiensis*, *C. spicifera,* and *E. rostratum,* respectively, for the strains ML9021, PK9021, FR9030, and BF9006. Sequences were deposited in NCBI GenBank under accession numbers OP554702, OP554633, OP473603, OP473604, OP473605, OP554576, OP554691, and OP473583. For whole-genome sequencing, each isolate was cultured for 7 days at 25°C (12 h photoperiod) using the Corn Meat Agar (18 g/L) medium previously covered with a sterile cellophane disc. DNA was extracted using approximately 60 mg of fresh mycelium using the cetyl trimethyl ammonium bromide method (8). The genomic library was prepared using the TruSeq Nano DNA library preparation kit from Illumina. Pair-end sequencing (2 × 150 pb) was performed with an Illumina NovaSeq 6000, with an average depth of 50×. The demultiplexing and the production of the fastq files were performed using the bcl2fastq software v2.20.0.422. The quality of sequencing reads was determined using the FastQC program (http://www.bioinformatics.babraham.ac.uk/projects/fastqc/) and FastQ Screen (https://www.bioinformatics.babraham.ac.uk/projects/fastq_screen/) especially for contaminant detection. Assembly was performed with ABySS software v2.2.1 (9) for different kmer values, and genome completeness was evaluated using BUSCO v5.4.3 with the Pleosporales lineage data set (6,641 genes) (10).

**Fig 1:**
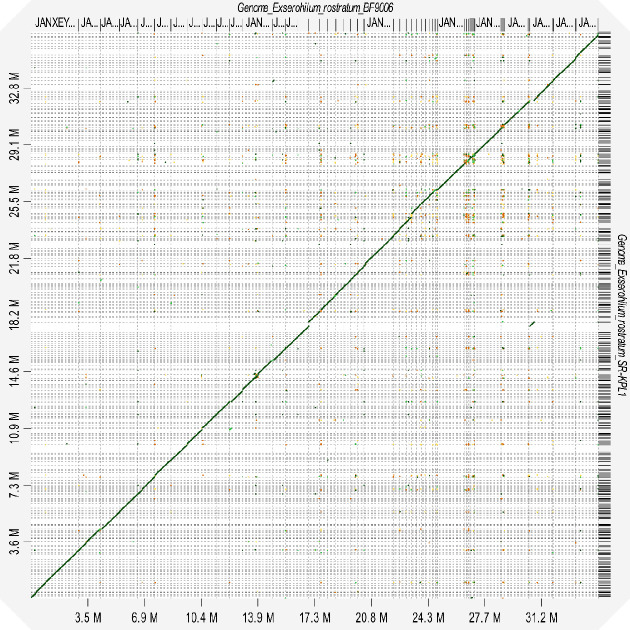
Dot plot analysis for genome comparison of *E. rostratum* strains BF9006 and SR-KPL1 using D-GENIES (13). BF9006 as a target is on the x-axis, and SR-KPL1 as a query is on the y-axis. Genomic alignment regions are presented as four-colored lines, corresponding to different similarity values (Yellow: <25% similarity, Orange: 25–50% similarity, Green: 50–75% similarity, and Dark Green: >75%). The long stretches of dark green lines in the diagonal indicate high nucleotide similarity between the two strains of *E. rostratum*.

Total genome lengths ranged from 30 to 35 Mb, the number of scaffolds ranged from 99 to 412, and GC content ranged from 49.14 to 51.17% (Table 1). The BUSCO evaluation showed that the assembly recovered over 93% of the expected single-copy genes in the Pleosporales order. Compared to the *E. rostratum* genome of strain SR-KPL1 isolated from rice in India (11), strain BF9006 described in this study has fewer scaffolds (99 versus 769) and a larger N50 value (340,895 versus 122,824 bp) attesting data quality. The average nucleotide identity (ANI) analysis of the two complete genomes performed using pyani v0.2.11 (12) showed a similarity of 98.32%. Visualization of the alignment of the two genomes (Fig. 1) using D-GENIES v1.5.0 (13) corroborates the high ANI values.

**TABLE 1:** General features of the complete genome sequences of *Bi. bicolor, C. hawaiiensis*, *C. spicifera,* and *E. rostratum* isolated from rice

Sequencing		Assembly				BUSCO analysis				
Strains	Coverage depth (x)	No. of scaffolds	Size (bp)	GC %	N_50_ (bp)	% of complete	% of complete and single copy	% of complete and duplicated	% of fragmented	% of missing
*B. bicolor* strain ML9021	62.9	218	35,093,885	49.14	965,787	95.45	95.38	0.07	0.42	4.12
*C. hawaiiensis* strain PK9021	205.5	412	30,043,159	51.17	179,076	94.28	92.82	1.46	0.62	5.10
*C. spicifera* strain FR9030	69.4	171	31,738,811	50.98	745,506	94.85	94.64	0.21	0.39	4.80
*E. rostratum* strain BF9006	74	99	34,670,870	50.70	1,163,278	94.60	94.40	0.21	0.45	4.94

## Data Availability

Whole-genome sequences were deposited in NCBI/GenBank under accession numbers JAODYD000000000, JAODYC000000000, JAODYB000000000, and JANXEY000000000. The raw data were deposited in the NCBI Sequence Read Archive (SRA) under SRA accession number SRR23346300, SRR23346299, SRR23346298, and SRR22883102.
